# Rate-Dependent Tensile Behavior of Glass Fiber Composites Reinforced with Quadriaxial Fabrics, with or Without Coremat Xi3 Interlayer, for Marine Applications

**DOI:** 10.3390/polym17152074

**Published:** 2025-07-29

**Authors:** Lorena Deleanu, George Pelin, Ioana Gabriela Chiracu, Iulian Păduraru, Mario Constandache, George Ghiocel Ojoc, Alexandru Viorel Vasiliu

**Affiliations:** 1 Polymer Processing Research Center, “Dunarea de Jos” University, 111 Domneasca, 800201 Galati, Romaniamario.constandache@gmail.com (M.C.); 2 National Institute for Aerospace Research (INCAS) “Elie Carafoli”, 220 Iuliu Maniu, 061126 Bucharest, Romania; 3 Autonomous Flight Technologies, 1 Aeroportului, 077060 Clinceni, Romania; george.ojoc@gmail.com

**Keywords:** glass fiber composite, tensile test, test rate, quadriaxial fabric, epoxy resin, unsaturated polyester resin, Coremat Xi3

## Abstract

This study is among the first to characterize the tensile response of composites with quadriaxial glass fiber fabrics designed for marine structural applications. Four composite configurations were fabricated at laboratory scale, combining two matrix types (unsaturated polyester resin and epoxy resin) and the presence or absence of a Coremat Xi3 middle layer. Tensile tests were conducted at four test rates (10 mm/min, 200 mm/min, 500 mm/min, and 1000 mm/min), ranging from quasi-static to moderately dynamic conditions. Tests were conducted using the Instron 5982 universal testing machine (from Laboratory for Advanced Materials and Tribology, INCAS Bucharest, Romania). The specimens have a rectangular cross section, in agreement with SR EN ISO 527-4:2023. For strain measurements, an Instron advanced video extensometer (AVE) was used. Key mechanical parameters, such as maximum force, tensile strength, Young’s modulus, strain at break, and energy absorption, were extracted and analyzed. Results show that the polyester-based composite without a mat interlayer displayed the best overall performance, with the highest ultimate strength (~280 MPa), significant energy absorption (~106 J), and a consistent increase in ductility with increasing test rate. In contrast, the epoxy composite with Coremat Xi3 exhibited lower stiffness and strength, but higher strain and energy absorption at higher test rates, indicating a progressive failure behavior. These findings enhance the understanding of the tensile response of composites made of quadriaxial glass fiber fabric and provide valuable design data for structural components in marine environments, where both strength and energy absorption are essential. These insights support producers and end-users of non-crimp fabrics in making experimentally based selections of a composite, technological strategies, and design optimization.

## 1. Introduction

Tensile tests are essential for characterization of composite materials, especially for unidirectional and multidirectional composites, for the following reasons: determination of basic mechanical properties, ease of information comparison to other materials, assessment of batch quality according to the datasheet of the product, practical importance in design, and validation of simulation models and prototypes.

Even if fibers from natural, sustainable resources have been introduced in particular applications [1], the marine industry relies on glass, carbon, and synthetic polymer fibers as reinforcement [2,3], due to their set or mechanical and thermal properties, their robust design based on experimental results with high repeatability, and their durability in a severe environment [4].

Tensile tests provide direct information about tensile strength, defined as the maximum stress before failure, longitudinal modulus of elasticity that reflects the stiffness of the composite in a certain direction related to reinforcement, and elongation at break as the strain before failure. The curve of such characteristics drawn against time or displacement could give information on elastic and plastic behavior, which is important for structural analysis, especially for assessing the safety coefficient.

A structural design could be engineered using data obtained from tensile tests. These could be introduced in finite element simulation as constitutive material models and in failure criteria [5,6,7,8].

Commercial and academic documentation is sparse for quadriaxial fabrics included in composites, and the experimental results presented here could be considered a reference point for future studies.

Caramatescu et al. [9,10] have tested composite structures with different types of materials, including woven glass fiber fabric (WR) and mats with glass fiber whiskers (100 mm to 120 mm) (CSM). The resulting composites have thicknesses of 4.20 mm (2 layers CSM + 1 layer WR + 1 layer CSM for deck),, 4.90 mm (3 layers CSM + 1 layer WR + 1 layer CSM for sideboards), 6.20 mm (2 layers CSM + 1 layer WR + 1 layer CSM + 1 layer WR + 1 layer for bottom side) and 8.30 mm for keel (3 layers CSM + 1 layer WR + 1 layer CSM + 1 layer WR + 2 layers CSM). The matrix was an unsaturated polyester resin. The structure with only glass fiber fabrics attends a yield limit in traction of 131 MPa, for an approx. sample thickness of 5 mm. The authors proposed sequential arrangements using mats with glass fiber whiskers (CSM, with area density of 450 g/m^2^ and 600 g/m^2^), two woven fabrics (WR) with area density of 300 g/m^2^ and 500 g/m^2^, a PVC foam (grade M 55 SCRIM, supplied by Cristex), and a Coremat Xi (from Lantor BV) similar to that used in this research. The proposed solutions were evaluating by taking into account a set of characteristics, and their ranking placed the hybrid composite on the first line (1 layer CSM + 1 layer WR + 1 layer CSM +1 layer PVC foam + 1 layer CSM + 1 layer Coremat Xi +1 layer CSM + 1 layer WR + 1 layer CSM). The lowest displacements (by 44.8%) and the lowest stress (by 29.7%) were calculated, compared to the original variant, having the only disadvantage of a larger mass (but with only 8.6%).

Rajak D. K. et al. [11] reported characteristics in bending and traction for fiber reinforced epoxy composites used in car body parts, with and without Co filler (0.4 to 1.0 wt.%). The glass fiber composites have tensile and flexural limits of approx. 97 MPa and 120 MPa, respectively, while tensile and flexural limits of composites with carbon fibers were approx. 195 MPa and 393 MPa, respectively. Glass fiber composites have a thickness of 2.81–2.88 mm, and the carbon fiber composites have a thickness of 2.90–3.03 mm, all with a sample width of 15 mm.

Itu C. et al. [12] reported characteristics obtained in traction tests for a composite with glass fiber and epoxy resin. The composite could be considered as hybrid as its architecture is OSB (middle layer of thickness of 10 mm), MAT 450 (2 layers of thickness of 2 mm), and chopped glass fiber with resin (2 layers of thickness of 2 mm), resulting in 18 mm for the composite. The tensile tests of each component allow for simulating the behavior of the stratified composite, but tests on actual composite were not reported.

Cao Y. et al. [13] tested composites with two, four, and eight layers of glass fiber fabric (with 0.2 mm as thickness and 200 g/m^2^ as areal density). Comparing tensile characteristics from the tensile test, and the authors reported that the uneven loading between layers in the composite with eight layers caused internal delamination and uneven destruction, reducing the tensile properties of laminates. In addition to the effect of composite thickness, the epoxy resin systems and interfacial performance influenced the tensile properties of the elaborated composite. Using different epoxy resins (with ultimate tensile strength from 25 MPa to 41 MPa), the same characteristic of the four-layer composite varied from 95 MPa to 215 MPa, respectively. For these composites, the increase in resin tensile strength induced higher values for the ultimate tensile strength of the four-layer composite from 98 MPa to 215 MPa. Details of fabric architecture and glass fibers’ quality are not given.

Applications of glass fiber composites include components in renewable energy (wind turbine blades, mainly) [14], marine industry [15], sport and leisure industry [16], automotive [17], aerospace [18], and military applications [19].

Table 1 presents recent research studies on glass fiber composites and points out the diversity of the tested composites and also that thicker composites are not yet investigated even if industries ask for this information, especially in shipbuilding, aircraft and military applications.

Quadriaxial fabric is formed by reinforcement fibers, oriented in four directions, held in place by a secondary non-structural stitching thread. Its advantages include higher fatigue resistance, less resin consumption, better mechanical properties and fade-out of anisotropy, improved speed of fabrication of a product, better drapability, and higher delamination resistance and impact strength. They are widely used in high-performance sectors (marine, wind energy, automotive), but rarely explored in deep research. Continuing our previous work, where the mechanical behavior of the studied glass fiber composites was assessed using bending and impact tests, this paper presents the results of tensile testing on the same composite materials. By expanding the range of mechanical characterizations across different loading conditions, we aim to provide a more comprehensive understanding of material behavior. Such a multifaceted approach supports more reliable structural design and enhances the safety assessment of composite structures. Thus, our experimental campaign could fill a gap between industry practice and laboratory tests, based on new entry composites, such as those having quadriaxial fabrics as reinforcement. For these composites, the experimental results will be fundamental for designing new components. As non-crimp fabrics become more common in structural composites, our paper may become foundational for design engineers and researchers alike.

While the first producer of quadriaxial fabrics is challenging to be established, several companies were involved in the early development and trade of quadriaxial fabrics, including Saertex—a German company recognized for pioneering multiaxial non-crimp fabrics, including quadriaxial variants [31]; Metyx Composites [32]; Solidian-Kelteks [33]; and Castro Composites [34].

The adoption of quadriaxial fabrics has been particularly prominent in industries such as marine, wind energy, automotive, and aerospace, where the materials’ balanced mechanical properties and processing advantages are highly valued.

Despite their growing use, limited data exist regarding the mechanical performance of quadriaxial fabric composites under various loading conditions. Most of the literature focuses on unidirectional or bidirectional reinforcements, often excluding multi-axial architectures. This study aims to bridge that gap by analyzing four specific composite structures and their tensile response as a function of test rate and composite constituents. Particular attention is paid to the influence of resin type and the presence of interlayers, which are critical factors in marine applications where strength, stiffness, and damage tolerance must be finely balanced.

## 2. Materials and Methods

The same composites were tested in bending and under impact in previous papers [22,35], with details on the laboratory-scale production. Thus, here are briefly described the materials used for producing the composites and their fabrication. It is interesting to mention that, even if we obtained four different composites, the manufacturing operations at laboratory scale are the same. Thus, the producers could use the same equipment for a larger product range.

**Figure 1:**
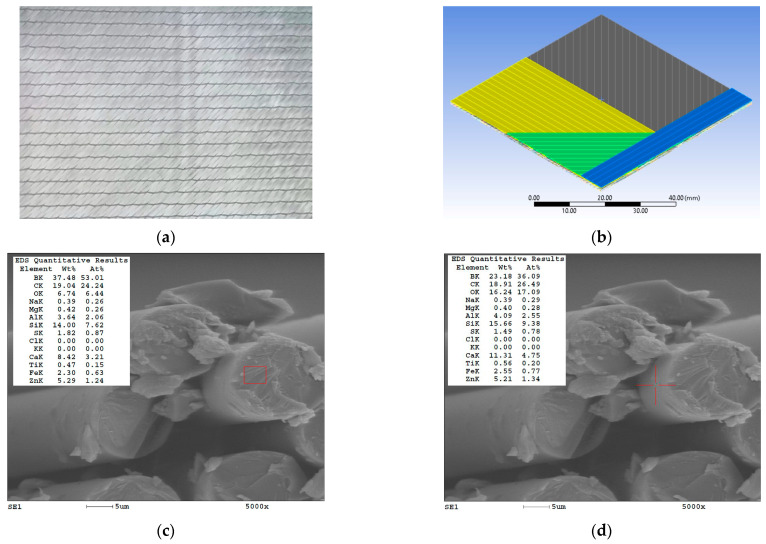
Quadriaxial fabric: (**a**) view from the Castro Composites catalog [32]; (**b**) a model of the sub-layer arrangement (only several yarns are visible in order to point out their positions): 0° (blue yarns), −45° (green yarns), 90 (yellow yarns), and −45° (grey yarns); (**c**) one of the EDX analyses for the core of the glass fiber (analyzed zone is the red rectangular shape), at magnification × 5000; (**d**) one of the EDX analyses for the jacket of the glass fiber (analyzed zone is a punctual one, noticed by the red cross lines), at magnification × 5000.

The reinforcement is the same, quadriaxial glass fiber fabric, supplied by Castro Composites (Porriño, Pontevedra, España); the yarns’ arrangement is 0°/+45°/90°/−45° [36]. Figure 1 presents aspect of the quadriaxial fabric: (a) a view as given in the producer catalog [36], (b) a model of the yarns’ arrangement on the four sub-layers with different orientations, (c) the EDX analysis of the glass fiber core (in the red area), and (d) the EDX analysis of the glass fiber jacket (in the red indicated point). These EDX analyses were repeated for the core and the jacket of several glass fibers in order to obtained an average, with glass fibers from the same fabric being analyzed in [37]. Fiber diameter ranges between 11 μm to 17 μm, as presented by the producer [36]. The SEM images were obtained with the help of FEI Quanta 200 scanning electron microscope from “Dunarea de Jos” University (supplied by FEI Company, Hillsboro, OR, USA, now Thermo Fisher Scientific Inc.) and its software includes an analysis software with EDX spectrometer (namely EDAX32, Genesis software 5.10).

**Figure 2:**
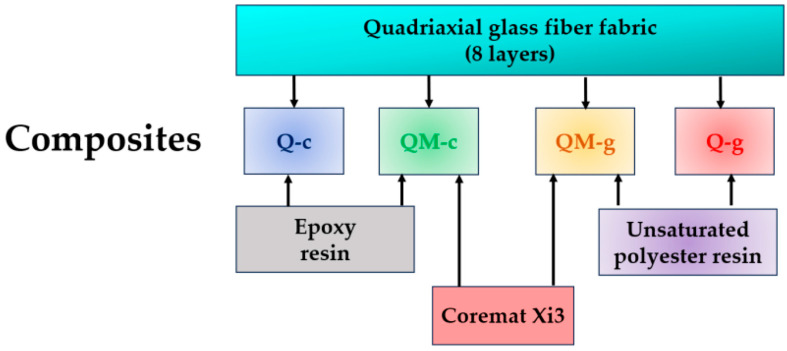
Chart of composite components and the codification colors for each composite is kept in the figures with experimental data.

Figure 2 presents the components of the four composites tested under traction. The reinforcement is the same, quadriaxial glass fiber fabric supplied by Castro Composites (Porriño, Pontevedra, España); the yarns’ arrangement is 0°/+45°/90°/−45° [32].

We selected Coremat^®^ Xi 3 mm (from Lantor BV, Veenendaal, The Netherlands), supplied by Rompolimer SA (Bucharest, Romania) [38,39,40,41]. This is used as a core material for ships, automotive components, leisure and sport components, or industrial structures because its set of properties is acceptable for taking into account criteria such as strength, flexibility, and aesthetics. It is made of non-woven polyester fibers with integrated microspheres and is compatible with resins such as epoxy resins, unsaturated polyesters, vinyl esters, and phenolics [38,39].

“Coremat^®^ is made by incorporating microspheres in a polyester based fleece. Because of these microspheres, Coremat^®^ creates a low weight volume and increases stiffness without the extensive use of costly materials” [41]. Several characteristics are described as follows: dry weight of 80 g/m^2^, impregnated density of 630 kg/m^3^. Typical mechanical properties of Lantor Coremat^®^ Xi (3 mm) impregnated with unsaturated polyester resin [38] are a flexural modulus of 1.1 GPa and flexural strength of 11 MPa. The resin is well impregnated in Coremat^®^ Xi 3, and the quantity is relatively low (approx. 600 g/m^2^ for 1 mm of thickness of the resulting composite).

We selected two resins based on producers’ documentation [42,43], namely, an epoxy one and an unsaturated polyester resin. Both are briefly presented.

The two-component epoxy resin SikaBiresin^®^ CR82 with the hardener CH80-2 is supplied by the manufacturer Sika Group (Sika Österreich GmbH, Bludenz, Austria) [44] through PolyChem Chemical SRL, Bucharest, Romania. The curing cycle, applied after seven days of natural aging was carried out at 60 °C for 6 h, followed by cooling at a rate of ~0.5 °C/min.

The other resin was the unsaturated polyester resin Enydyne H 68372 TA (from Polynt Composites, Niepołomice, Poland) [45,46,47] with Metox-50W [48] as the accelerator supplied by Rompolimer Composites [49].

The quadriaxial glass fiber fabric was cut, and a set of 8 layers were weighted; the mold was coated with CIREX CP 10, an extraction wax (producer Airétec [50,51], distributor RomPolimer Composites SA, Bucharest, Romania) used for polyester and epoxy resins. It is a soft release agent that evaporates at room temperature and forms a thin monomolecular layer that adheres to the mold walls and has no affinity for the resin or the composite. The composites were laying up with resin, layer by layer, pressed in mold, and we controlled the thickness with the help with a multi-screw device, also presented in [37].

The Coremat Xi3 layer was also laid up with resin in the same way. Then, a uniform pressure was applied (0.5–0.7 MPa) on the composite for at least 8 h. All fabricated composite plates can be removed from the mold at room temperature.

**Figure 3:**
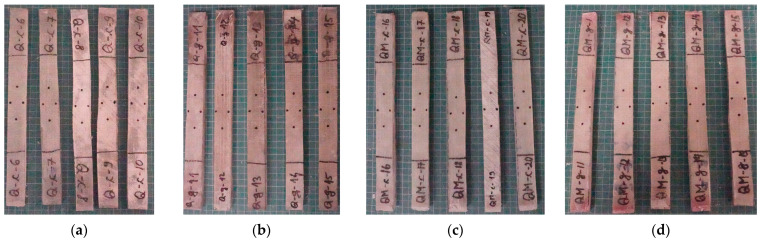
Sets of five composite specimens, with marks for monitoring of strain with the help of AVE system: (**a**) Q-c (unsaturated polyester resin); (**b**) Q-g (epoxy resin): (**c**) QM-c (unsaturated polyester resin + 1 middle layer of Coremat Xi3): (**d**) QM-g (epoxy resin + 1 middle layer of Coremat Xi3). Each composite has eight layers of quadriaxial glass fiber fabric.

Tensile tests were carried out on the Instron 5982 universal test machine [52] (from INCAS, Bucharest, Romania). The machine was equipped with a 100 kN force cell, with dedicated software [53], and special grips 2736-004 for tensile tests [54], with a load measurement accuracy to +/−0.5% of reading down to 1/1000 of load cell capacity. For strain measurements, an Instron advanced video extensometer (AVE) [55] was used. Figure 3 presents a set of five test samples, for each composite, with marks for using the extensometer.

Information on fabricated plates are given in Appendix B. One may notice the very small variations of the parameters in Table A1 and Table A2.

ISO 527-1:2019 [56] recommends 13 values for the tensile test rate, starting with 0.125 mm/min, and the highest value is 500 mm/min. ISO 527-4:2021 [57] and ISO 527-5:2021 [58], standards that deal with the tensile testing of composites, refer to ISO 527-1 regarding the testing speed.

ASTM D3039/D3039M—17 Standard Test Method for Tensile Properties of Polymer Matrix Composite Materials [59] suggests a standard head displacement rate of 2 mm/min, but does not limit this parameter. It only specifies that sample failure should be in the range of 1–10 min. It is underlined that for quasi-isotropic laminates, such as our composites, the edge effects and ply orientations are not as significant. The constant rectangular cross-section is also promoted, with the length being 250 mm for either balanced and symmetric or randomly discontinuous arrangement of the reinforcement, and the thickness and width of the specimen could be “as needed”.

ISO 527-5:2021 [58] recommends a test speed for type A specimens of 2 mm/min and for type B specimens of 1 mm/min. For many components made of composites with polymeric matrix, these test speeds are too low as compared to the actual loading speed.

ISO 527-4:2021 [57] is in the favor of testing new composites, as it could be applied for the following:

- Fiber-reinforced thermosetting and thermoplastic composites incorporating non-unidirectional reinforcements such as mats, woven fabrics, woven rovings, chopped strands, combinations of such reinforcements, hybrids, rovings, short or milled fibers, or preimpregnated materials (prepregs);
- Combinations of the above with unidirectional reinforcements and multidirectional reinforced materials constructed from unidirectional layers, provided such laminates are symmetrical;
- Finished products made from materials mentioned above.

We cite this text from the standard in order to emphasis the very wide range of composite materials, innovative or “classic”, that could be tested following this standard procedure. However, this standard also recommends low test speeds: 10 mm/min for routine quality control for specimens 1B and 5 mm/min for specimen types 2, 3 and 4; a test speed of 2 mm/min for qualification tests for all specimens for qualification tests. Our samples are in agreement with sample type 3, without end tabs and with overall length 300 mm > L_3_ ≥ 250 mm and the initial distance between grips (nominal) of 150 mm. Table 2 presents the test rates the authors used in this study and the equivalent strain rates, calculated based on the length between the extensometer length L_0_ = 50 mm [55].

Typical tensile strain rates experienced by composites during various marine events—collisions and wave impacts are as following [60,61,62,63,64]:

- Global structural strain rates remain low, typically < 1 s^−1^, often 0.1–10 s^−1^;
- In a ship-side collision simulation, local equivalent plastic strain rates up to ~30 s^−1^ just before fracture, in a complex model [60];
- Low-velocity wave slamming is similar to quasi-static, with strain rates just a few s^−1^, often negligible change in response [62];
- Under more violent conditions (high-speed slamming or wave impact), strain rates can rise to tens or hundreds of s^−1^, though exact values vary with energy and structure;
- In extreme “blast-like” marine events, such as freak waves, strain rates can reach 1 s^−1^ to 10^4^ s^−1^, with an average around 10 s^−1^ near the blast range [63].

Taking into account the above mention values for the strain rate, the composite structures in marine applications should be tested at larger range for the strain rate; thus, this study offers information on a larger test rate interval for the mechanical characteristics.

ISO 527-1:2019 [56] recommends 13 values for the tensile test rate, starting with 0.125 mm/min and with the highest value being 500 mm/min. We selected four test rates, three are included in this range (10 mm/min, 200 mm/min and 500 mm/min) and one is higher (1000 mm/min), taking into account the machine test parameters and the above discussion.

## 3. Results

**Figure 4:**
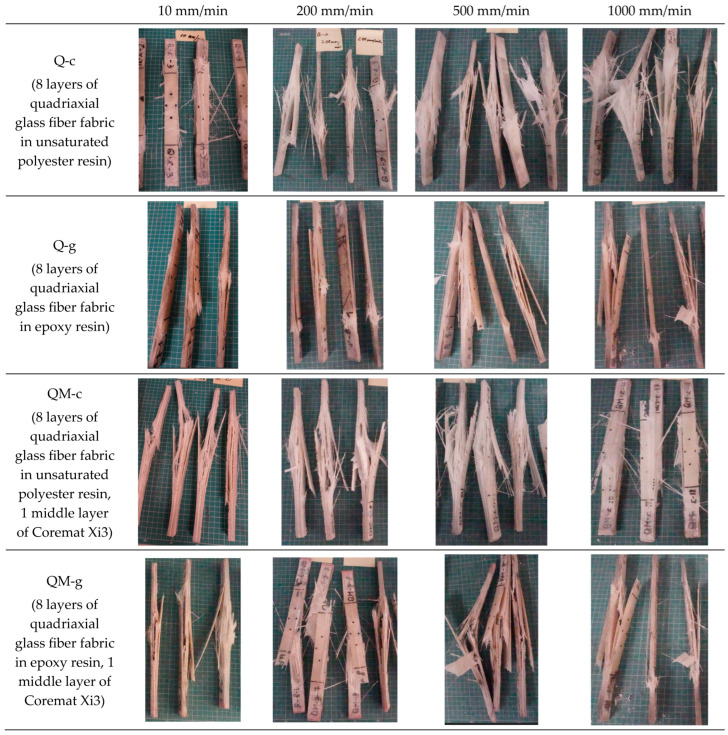
Failure aspects of the tested composites, organized based on composite material (lines) and test rate (column).

Figure 4 presents typical failure aspects of each tested composite and for each test rate. Progressive damage is noted as the test rate increases. All composites show increasing damage severity (more fraying, fiber pull-out, delamination) as the test rate rises from 10 mm/min to 1000 mm/min. These photos sustain a qualitative evaluation of these composites, suggesting that all materials are sensitive to strain rate to some extent. At higher strain rates (500 mm/min and 1000 mm/min), fiber breakage dominates across all composites, indicating the transition from matrix-dominated to fiber-dominated failure behavior. In both QM-c and QM-g composites, failure tends to initiate or localize around the Coremat Xi3 interface, implying that this internal layer systematically influences crack propagation, and it is less resistant than the stratified structure (composite glass fibers + resin matrix) near it.

At the highest test rate (1000 mm/min), the composite Q-g presents random delamination and is less predictable. The composite QM-g has the most chaotic failure among all, and failure aspects are highly variable, with unstable crack paths.

The composite Q-c seems to have the more consistent damage uniformity across test rates.

The experimental results reveal that the matrix–reinforcement interaction plays a crucial role in tensile performance. The polyester resin-based composite (Q-c) demonstrated the most favorable balance among strength, ductility, and repeatability, making it the best performer among these four tested composites, across multiple criteria. The addition of the Coremat Xi3 layer (in the middle of the composite) generally improved strain at break and energy absorption, but at the cost of stiffness and tensile strength, especially in the epoxy system. These trends confirm that the choice of matrix and internal layering must be tailored according to application needs—whether rigidity or flexibility is prioritized. Moreover, the reduced test rate sensitivity, except the passing from the lowest test rate (10 mm/min) to 200 mm/min, observed for all composites, suggests that test rate is a factor that could be approximated as constant for the tested range, even if this is a non-negligible factor in material selection for real-world dynamic loads.

Based on the photos given in Figure 3, we summarized our observations taking into account two criteria: the presence or absence of mat (Table 3) and the test rate for each composite (Table 4).

**Figure 5:**
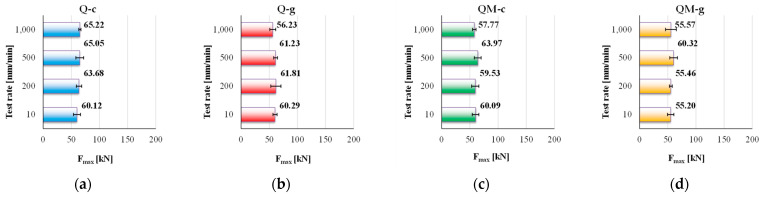
The values of F_max_ (as an average value of five tests with standard deviation) for the composites: (**a**) Q-c; (**b**) Q-g; (**c**) QM-c; (**d**) QM-g.

Figure 5 presents the maximum values of measured force, F_max_, for each composite, as the average of five tests and the standard deviation of the measurements. The maximum tensile force values (F_max_), determined as the average of five tests for each composite and testing rate, highlight distinct behaviors depending on the resin type and the presence or absence of the Coremat Xi3 interlayer.

The analysis of the maximum tensile force values (F_max_) reveals a variation on a quite small range (55.2 KN to 65.22 kN, a variation of 15.36% reported to higher value), with the common constituent being the glass fiber fabric. This means that, under traction, the fibers would “dictate” the value of these characteristics, even if structure is different and the matrix resins are different.

Peak values, such as 65.22 kN for Q-c, at 1000 mm/min, and minimum value of 55.20 kN for QM-g, reflect a complex mechanical behavior. The polyester-based system proves efficient in the absence of a middle layer, while the combination of epoxy resin and Coremat Xi3 significantly reduces the load-bearing capacity. These findings are crucial for material selection in dynamic structural applications.

The Q-c composite (polyester matrix, without Coremat Xi3) exhibits the highest F_max_ value, reaching 65.22 kN, for the test rate of 1000 mm/min. A light upward trend is observed for this composite, starting from 60.12 kN, at 10 mm/min, indicating good mechanical adaptability to dynamic loading. This is notable given that polyester resins are generally considered less performant than epoxies, but these results suggest excellent compatibility between this matrix and the quadriaxial reinforcement, when loading in traction.

In contrast, the Q-g composite (epoxy matrix, without Coremat Xi3) shows a different trend: after a slight increase to 61.81 kN, at 200 mm/min, the values begin to drop, reaching 56.23 kN at 1000 mm/min. This significant decrease at a high test rate may indicate increased sensitivity of the epoxy matrix to fast loading, in the absence of a stress-diffusing interlayer.

The QM-c composite (polyester matrix with Coremat Xi3) presents a more distinguished evolution: starting from 60.09 kN, it rises to 63.97 kN at 500 mm/min, then decreases again to 57.77 kN, at 1000 mm/min. This fluctuation suggests that while the Coremat Xi3 layer may positively contribute at moderate test rates, it introduces a damping effect that limits the maximum value of the load under higher rates of deformation.

The QM-g composite (epoxy matrix with Coremat Xi3) shows the lowest values for F_max_ overall, with values ranging between 55.20 kN and 60.32 kN, with low variation. This suggests that the simultaneous presence of a stiff matrix and a soft, porous interlayer leads to an antagonistic effect that reduces the overall supported load.

In conclusion, the Q-c composite, with a polyester matrix and no interlayer, proved to be the most performant with regard to the maximum supported load, under increasing test rate. It combines high resistance with a stable growth trend of maximum tensile force, making it a suitable choice for structural applications involving dynamic loading. On the opposite end, QM-g displayed the weakest performance, suggesting that the addition of Coremat Xi3 in conjunction with an epoxy matrix may be detrimental in terms of the bearing tensile load. These results are highly relevant for material selection in structural applications subjected to variable or dynamic loading, where mechanical resistance under fast testing conditions is critical.

**Figure 6:**
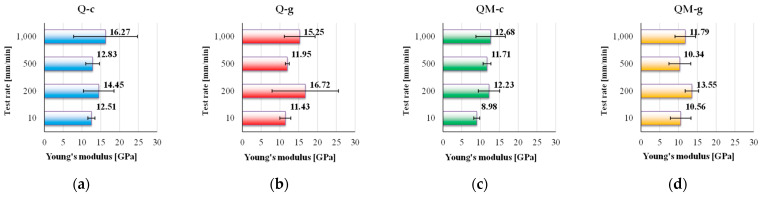
The values of Young’s modulus (as an average value of five tests with standard deviation) for the following composites: (**a**) Q-c, (**b**) Q-g); (**c**) QM-c); (**d**) QM-g.

Figure 6 presents the values for Young’s modulus, calculated for each composite. In terms of stiffness, the two composites without the interlayer (Q-c and Q-g) exhibited the highest Young’s modulus values, with a peak of 16.72 GPa for Q-g. However, Q-c stood out for its consistent increase in stiffness with test rate, suggesting better adaptability under dynamic loads. The Coremat Xi3-containing composites recorded lower values, between 8.98 GPa and 13.55 GPa, indicating a mechanical decoupling effect, introduced by the porous middle layer.

Young’s modulus provides essential information about the stiffness of composite materials in the linear elastic regime. It reflects the material’s ability to resist deformation under axial loading and is critical in structural applications where dimensional stability is required. It plays a critical role in the design of composite structures. Ideally, structural components should be designed using multiple modulus values corresponding to different loading rates, especially when dynamic effects are expected.

However, in this study, the experimental results reveal a limited sensitivity of Young’s modulus to the test rate. This finding, based on experimental results, significantly simplifies the design process. The designer may either adopt the most conservative (the lowest) value or apply a calibrated safety factor to cover minor rate-induced variations.

This stability is particularly advantageous for real-world applications where loading rates may vary, such as in marine structures exposed to wave impacts, vibration, or fluctuating operational loads.

From a modeling and simulation perspective, the rate-dependent values of Young’s modulus can be used to define more accurate material constitutive models, particularly under dynamic or transient loading conditions.

However, the limited sensitivity of Young’s modulus to test rate observed in this study allows the use of a constant elastic modulus in numerical models. This simplifies structural analysis and enables reliable predictions without the need for complex viscoelastic or strain rate–dependent formulations.

The Q-c composite (polyester matrix, without Coremat) shows an increasing trend for Young’s modulus, with values ranging from 12.51 GPa at 10 mm/min to 16.27 GPa at 1000 mm/min, demonstrating a consistent increase in stiffness with test rate. This sustained growth suggests a strong interface between matrix and reinforcement and an efficient load transfer capacity under dynamic conditions.

The Q-g composite (epoxy matrix, without Coremat Xi3) achieves the highest overall value (16.72 GPa at 200 mm/min) but exhibits significant variation, starting from 11.43 GPa at 10 mm/min, and reaching 15.25 GPa, at 1000 mm/min. This irregular trend may indicate a sensitivity of the epoxy matrix to low-rate testing, possibly due to early microcracking or incomplete stress relaxation.

The QM-c composite (polyester with Coremat Xi3) has lower stiffness overall, with a minimum of 8.98 GPa at 10 mm/min and a maximum value of 12.68 GPa, at 1000 mm/min. The presence of the Coremat Xi3 layer appears to reduce overall rigidity, although its influence diminishes at higher test rates, possibly due to local compaction of the intermediate layer.

QM-g (epoxy with Coremat Xi3) shows a wavy behavior (in a band of 3 GPa), ranging between 10.56 GPa and 13.55 GPa, with the highest value at 200 mm/min. Despite the inherent rigidity of epoxy resin, the Coremat Xi3 layer seems to limit its effect under extreme test rates, likely due to partial mechanical decoupling at the porous interface.

Overall, Q-g (epoxy matrix, no Coremat Xi3 layer) provides the highest peak stiffness (or Young’s modulus), but Q-c (polyester resin as matrix, no Coremat Xi3 layer) demonstrates the most stable and predictable growth with test rate, making it mechanically robust under variable loading conditions. Both Coremat Xi3-containing composites display significantly reduced values of Young’s modulus, confirming this interlayer’s negative effect on the elastic load-bearing capacity.

**Figure 7:**
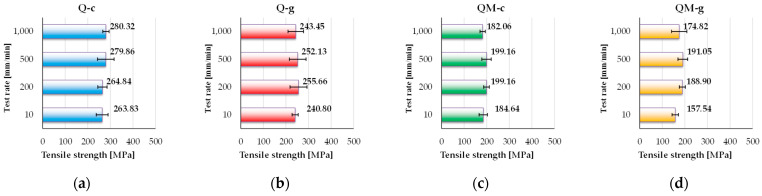
The values of tensile strength (as an average value of five tests with standard deviation) for the composites: (**a**) Q-c; (**b**) Q-g; (**c**) QM-c; (**d**) QM-g.

Figure 7 presents the ultimate tensile strength for each composite (tensile stress calculated for F_max_). Tensile strength, expressed in MPa, is a key parameter for evaluating the mechanical performance of composite materials in structural applications. It defines the maximum axial stress a material can withstand before failure and reflects the effectiveness of the reinforcement–matrix interface as well as the overall structural behavior under static or dynamic loading.

Analyzing these values, one may notice differences between the two composites without Coremat Xi3 (Q-c and Q-g) and those with a middle layer of Coremat Xi3, for which the tensile strength is much lower. However, all composites have a reduced sensitivity of this characteristic to the test rate. The tensile strength results emphasize the superior performance of the Q-c composite (peak of 280.32 MPa), followed by Q-g (~255 MPa). The presence of Coremat Xi3 clearly lowers this value, especially for the composite QM-g (minimum of 157.54 MPa). These findings indicate that, for marine or structural applications, mat-free composites are better suited in terms of axial mechanical resistance.

The Q-c composite (polyester matrix, without Coremat Xi3) exhibits the highest tensile strength values, with a peak of 280.32 MPa, at 1000 mm/min and a minimum of 263.83 MPa at 10 mm/min. A consistent increase is observed with rising test rate, indicating a favorable structural response under dynamic loading and a strong compatibility between the polyester matrix and the quadriaxial glass fiber reinforcement.

The Q-g composite (epoxy matrix, without Coremat Xi3) shows slightly lower values, ranging between 240.80 MPa and 255.66 MPa, with minor fluctuations depending on test rate. Although epoxy is generally known for superior mechanical properties, these results suggest that, in the absence of a core layer, polyester resin may perform comparably or even better under certain test conditions.

For QM-c (polyester matrix with Coremat Xi3), the tensile strength values are significantly lower, between 187.64 MPa and 199.16 MPa. While there is a slight increase up to 500 mm/min, a drop to 182.06 MPa, at 1000 mm/min indicates that the presence of Coremat Xi3 negatively affects performance at higher test rates.

QM-g (epoxy resin with Coremat Xi3) performs the worst among all tested composites, with tensile strength values ranging from 157.54 MPa to 191.05 MPa. Although it peaks at 500 mm/min, the value drops again to 174.82 MPa at 1000 mm/min, suggesting a loss of stiffness caused by local decoupling effects introduced by the porous interlayer.

In conclusion, the experimental results clearly demonstrate that the presence of Coremat Xi3 significantly reduces tensile strength, regardless of the matrix type. Q-c remains the top-performing composite configuration and is recommended for structural applications where axial load-bearing capacity is a key criterion. In contrast, QM-g should be avoided in such contexts, due to its limited performance, likely resulting from the combination of a stiff matrix with a compliant (but not very resistant) core structure.

**Figure 8:**
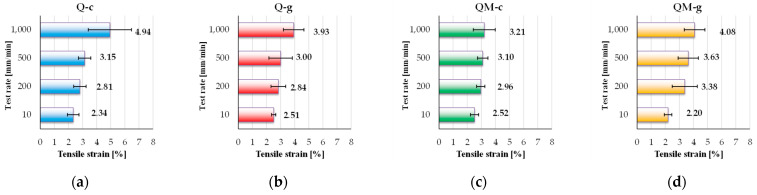
The values of tensile strain at break (as an average value of five tests with standard deviation) for the following composites: (**a**) Q-c, (**b**) Q-g; (**c**) QM-c; (**d**) QM-g.

Figure 8 presents the tensile strain at break, calculated for each composite. The tensile strain at break, expressed as a percentage, is a key indicator of a composite material’s ductility. It reflects the material’s ability to undergo deformation prior to failure and provides insight into its pseudo-plastic behavior and energy absorption capacity under tensile stress. Analyzing these values, one may notice a small sensitivity to the test rate: all composites have the smallest value for the lowest test rate (10 mm/min) and the highest value for the highest test rate (1000 mm/min). The strain at break shows a clear tendency to increase with test rate, with the highest value (4.94%) recorded for Q-c. Despite having a high-quality epoxy matrix, QM-g reaches 4.08%, at 1000 mm/min, demonstrating pseudo-plastic behavior induced by the Coremat Xi3 layer. Such a response may be valuable in applications where flexibility is critical. Monitoring critical zones of the structure (as a hull of a ship) (presumed/calculated to have higher strains) and comparing with admissible values for strain depending on elastic region in the stress–strain curve could avoid catastrophic events or could impose a preventive maintenance or a change in operational conditions. Monitoring strain levels and comparing them with the strain at break allows early detection of fatigue, prompting repairs before failure occurs.

In addition, knowing the strain at break allows engineers to assess how large deformation of a structural component (like a hull or a deck) can withstand before failure, which is crucial during storms or collisions. Materials with high strain at break are preferred for flexible joints or hull sections that must absorb energy during impacts without cracking. Understanding and knowing the strain at break helps avoid sudden brittle failure, which in marine environments could lead to flooding, loss of structural integrity, or sinking.

A shipboard engineer could monitor two key deformation zones:

- Strain up to the elastic limit, which corresponds to normal operating conditions, where the structure returns to its original shape after the load is removed;
- Strain beyond the elastic limit, but below the strain at break, indicating that the material has entered the (elasto-)plastic deformation range and is experiencing advanced loading.

By continuously comparing measured strain values (from onboard strain gauges or other sensors) to these thresholds, engineers can make informed decisions—such as scheduling preventive maintenance or altering the ship’s operation (e.g., reducing speed, changing course, or avoiding severe weather)—in order to avoid approaching failure and prevent catastrophic damage.

The Q-c composite (polyester matrix, without Coremat Xi3) exhibits the widest variation with test rate, ranging from 2.34% at 10 mm/min to 4.94%, at 1000 mm/min. This notable increase suggests a pronounced viscoelastic behavior of the polyester matrix, which becomes more flexible under dynamic loading conditions.

The Q-g composite (epoxy matrix, without Coremat Xi3) shows a more uniform response, with strain values between 2.51% and 3.93%.

Although both resins have close values for the flexural modulus (3.3 GPa for Enydyne H 68372 TA and 3.0 GPa for SikaBiresin CH80-2), the tensile and flexural strengths are almost twice that for SikaBiresin CH80-2 than the resin Enydyne H 68372 TA. The resulting composites (Table 5), Q-c and Q-g as structures without a Coremat Xi3 layer, do not have such evident differences in mechanical characteristics, meaning that the reinforcement dominates these values and not the matrix. The same conclusion could be drawn for the two composites with Coremat Xi3. The middle layer lowered the tensile strength as compared to the composite without it, with almost 100 MPa for the composite having the polyester resin as matrix and with approx. 70 MPa for the composites with epoxy matrix. The conclusion from this discussion is that only experimental data of a composite should be relied on when designing or verifying a structure made a particular composite.

The QM-c composite (polyester with Coremat Xi3) maintains moderate and stable values: from 2.52% to 3.21%. The Coremat Xi3 layer appears to introduce a zone of controlled deformation, maintaining strain levels within predictable limits, but limiting the overall extensibility of the material.

The composite QM-g (epoxy resin with Coremat Xi3) shows the most interesting behavior: despite starting at 2.20% at 10 mm/min, it reaches 4.08%, at 1000 mm/min. This substantial increase suggests that the Coremat layer Xi3 acts as a tension absorber, partially offsetting the epoxy matrix’s rigidity and allowing for greater deformation under dynamic conditions.

In conclusion, Q-c demonstrates the highest strain capacity, while QM-g exhibits significantly increased ductility at higher test rates, which may be advantageous in applications involving impacts or vibrations. Conversely, Q-g and QM-c show a more rigid and rate-insensitive behavior.

**Figure 9:**
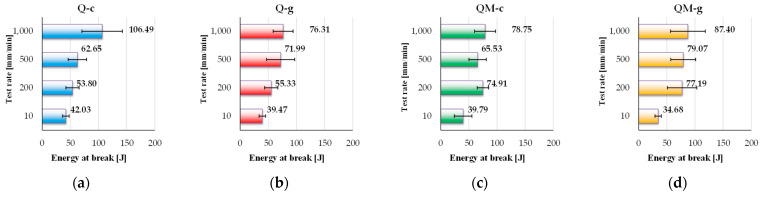
The values of energy at break (as an average value of five tests, with standard deviation) for the following composites: (**a**) Q-c; (**b**) Q-g; (**c**) QM-c; (**d**) QM-g.

Energy at break, measured in joules (J), represents the total amount of energy a composite material can absorb before complete failure. This parameter is crucial for evaluating the performance of composites in dynamic applications, such as impact-loaded structures, vibration-sensitive components, or cyclically loaded systems. Organizing the experimental data as in Figure 9, the differences among the four tested composites are pointed out.

Analyzing these values, one may notice a sensitivity to the test rate: all composites have the smallest value for the lowest test rate (10 mm/min) and the highest value for the highest test rate (1000 mm/min). For the lowest test rate (10 mm/min), the values do not differ too much; however, when the test rate increases, the composites with Coremat Xi3 have values of 74–77 J for composites QM-c and QM-g, at 1000 mm/min. The composites without a mat layer have a more differentiated trend. The composite Q-c has an average energy at break of 106.49 J, at 1000 mm/in, but Q-g has only 76.31 J for the same test rate. A beneficial contribution of the Coremat Xi3 layer is observed in QM-g and QM-c composites at higher speeds, suggesting high potential for applications requiring energy absorption/dissipation, such as marine structures exposed to impact or vibrations. It is worthy to point out that, for three of the composites (Q-g, QM-c and QM-g), the energy at break at 1000 mm/in is almost twice as the same characteristic at 10 mm/min. Only the composite Q-c has this ratio between energy at break for 1000 m/s and 10 mm/min of 2.5.

The Q-c composite (polyester resin, without Coremat Xi3) exhibits a clearly increasing trend, from 42.03 J at 10 mm/min to 106.49 J, at 1000 mm/min. This behavior highlights the polyester matrix’s efficiency in dissipating mechanical energy under fast loading conditions. The peak value of 106.49 J is the highest among all tested composites.

The Q-g composite (epoxy resin, without Coremat Xi3) shows relatively consistent behavior, with values ranging from 39.47 J to 76.31 J. Although the energy at break increases with test rate, the epoxy matrix appears to have a lower energy absorption capacity compared to the polyester one, likely due to its inherent stiffness and limited internal deformation mechanisms.

The composites that include the Coremat Xi3 interlayer—QM-c and QM-g—behave differently. At low speed (10 mm/min), their energy at break is modest (39.79 J for QM-c, 34.68 J for QM-g). However, as the test rate increases, both values rise significantly, reaching 78.75 J and 87.40 J, respectively, at 1000 mm/min. This trend suggests that the Coremat Xi3 layer plays an important role in additional energy dissipation, especially under dynamic conditions. Its local “damping” effect likely contributes to stress redistribution and progressive failure delay, enhancing the composites’ toughness.

In conclusion, Q-c shows the highest total energy absorption capacity, but QM-g and QM-c achieve comparable performance at higher test rates thanks to the presence of the Coremat Xi3 layer. Thus, these configurations are promising candidates for applications where impact resistance and energy dissipation are critical design factors.

**Figure 10:**
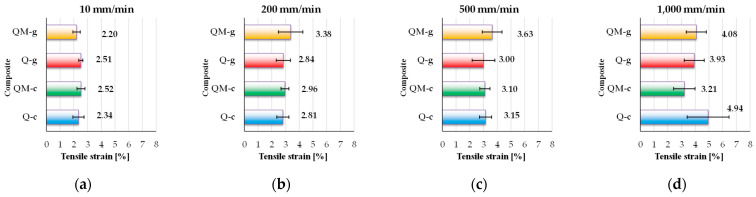
Experimental data (average values and standard deviations) for the strain at break at different test rates: (**a**) 10 mm/min; (**b**) 200 mm/min; (**c**) 500 mm/min; (**d**) 1000 mm/min.

Figure 10 presents the strain at break, a characteristic that proves to be less dependent on the composite architecture and has a slightly trend to increase with test rate, which is unusual for other materials like metallic alloys of polymers that commonly have smaller values for strain at break when the test rate increases.

Force–time, stress–strain, and strain–displacement curve details for all samples at various test rates are presented in Appendix A.

**Figure 11:**
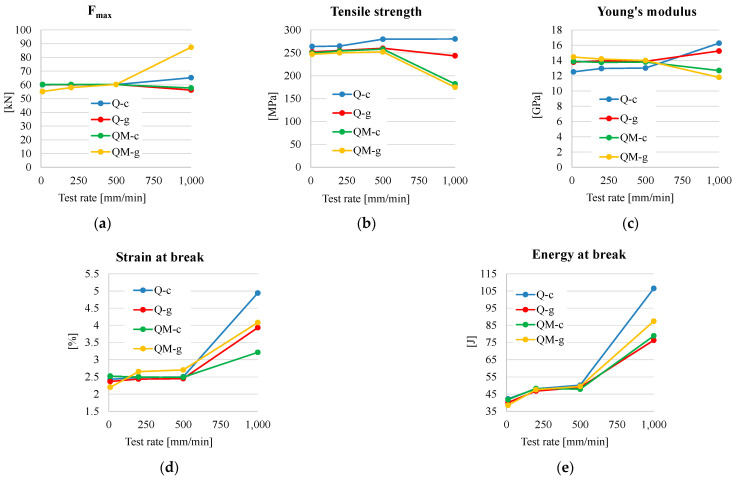
A synthesis of the experimental results based on average values of the characteristics determined from tensile tests: (**a**) F_max_; (**b**) tensile strength; (**c**) Young’s modulus; (**d**) strain at break; (**e**) energy at break.

Figure 11 summarizes the experimental results.

The measured values for F_max_ (Figure 11a) are grouped for the range 10–500 mm/min; however, for test rate of 1000 mm/min, this characteristic has a steeper slope for the composite QM-g, close to 90 KN. For the other composites, the evolution of Fmax is in a band of about 10 KN.

The dependency of tensile strength on test rate (Figure 11b) is different. In the range of a test rate of 10–1000 mm/min, composites without Coremat Xi3 have low sensitivity to test rate (only a very slight increase, for composite Q-c). The composites with Coremat Xi3 (QM-c and QM-g) have very close values for all test rates but very low ones at 1000 mm/min, with 182.06 MPa for QM-c and 174.82 MPa for QM-g. This is unusual behavior as other materials have an increase in these characteristics when the test rate increases.

Young’s modulus (Figure 11c) shows little increasing variation with test rate for composites without Coremat Xi3 and is more visible from 500 mm/min to 1000 mm/min, while this characteristic is decreasing obviously for the composites with Coremat Xi3 for the same interval of test rates.

The evolution of strain at break (Figure 11d) is not differentiated for the test rates 10–500 mm/min in a band limited by 2.25–2.75%. However, from 500 mm/min to 1000 mm/min, the strain at break increases visibly to 3.5% for QM-c to 5% for Q-c. This is an unusual behavior that could be explained by micro-local failures of matrix or/and fibers, without a total break of the composite sample.

The values of energy at break (Figure 11e) are well grouped for the test rates of 10–500 mm/min. The slope inclination is small, but the same slope between 500 mm/min and 1000 mm/min has a steeper inclination, which is favorable to absorb more energy in the case of a tensile loading. Further tests should be carried out for greater rates of loading. One may notice a sensitivity of energy at break to the test rate: all composites have the smallest value for the lowest test rate (10 mm/min) and the highest value for the highest test rate (1000 mm/min).

The rate-dependent trend of each tested composite could be pointed out by analyzing the variation of each characteristic, as reported at the value obtained at a test rate of 10 mm/min (Table 6).

Young’s modulus increases with test rate, but with 41.20% for the composite QM-c and only with 11.65% for composite QM-c. Composites without Coremat Xi3, Q-c and Q-g, have increases of 30.06% and 33.42%, respectively.

F_max_ has very small variations between the test rate 10 mm/min and 1000 mm/min, meaning that this characteristic is dependent on the reinforcement of the quadriaxial glass fiber fabric and not on the matrix type and the presence of Coremat Xi3 middle layer. Also, it reflects the quality of all fabricated plates.

Tensile strength, σ_max_, has also a small variation with test rate, except for composites Q-c and QM-g.

Strain at break increases for each composite, a more ductile behavior at 1000 mm/min is noted in composites Q-c and QM-g.

Energy at break is relevant for this test campaign. For each composite, this characteristic increases, with approx. 150% for Q-c and QM-g and only 93.34% and 97.91% for Q-g and QM-c, respectively.

Based on Table 6, the analyzed characteristics could be organized in two groups: Fmax and tensile strength with low sensibility to the test rate and the other group with characteristics increasing with the test rate, depending on the architecture and matrix resin of the composites. The authors emphasis that these conclusions are formulated and supported by experimental data only for the tested composites.

## 4. Conclusions

This study reports on characteristics obtained from tensile tests for composites designed by the authors and intended for marine applications. The common feature of all the four composites is the reinforcement made of quadriaxial glass fiber fabric. Two composites (Q-g and QM-g) have an epoxy resin as the matrix and the other two use an unsaturated polyester resin (Q-c and QM-c). Two composite types were fabricated with a middle layer of Coremat Xi3 (QM-c and QM-g).

We explored quadriaxial glass fiber composites—a reinforcement architecture underrepresented in the current literature, despite its growing use in industrial sectors like marine and wind energy. This study provides information on their behavior under tensile loading, and this information is useful for comparison with other glass fiber composites.

Through this tensile test campaign and mechanical characterization, we provide some of the first performance data for Castro Composites’ quadriaxial fabrics, which are usable in the marine industry (thicker composites). While unidirectional and bidirectional composites have been extensively studied, quadriaxial laminates lack experimental benchmarks. Our study begins to close that gap and to promote this type of composite due to its low sensitivity in traction to the test rate.

Our results offer designers a starting point to assess quadriaxial-reinforced composites, which are crucial for lightweight structural applications, but also for speed-up technological operations for composite components. These insights can inform producers and end-users of non-crimp fabrics, aiding value in selection, design, and optimization of components using this type of fabric.

The experimental analysis carried out on these composites types under uniaxial tensile loading conditions and varying test rates (10 mm/min to 1000 mm/min) revealed significant differences in mechanical behavior, which can be directly attributed to the matrix type (polyester vs. epoxy) and the presence or absence of the Coremat Xi3 interlayer. By correlating the data for several characteristics (maximum force, Young’s modulus, tensile strength, strain at break, and energy at break) with the detailed information provided by the force–time, stress–strain, and strain–time curves in Appendix A, a comprehensive and nuanced understanding of their tensile performance has been achieved.

Among all the materials tested, the Q-c composite (polyester matrix without Coremat) demonstrated the most balanced mechanical behavior, marked by consistently high tensile strength (~280 MPa at 1000 mm/min, with low variation with test rate), strong initial stiffness, and excellent repeatability among replicates. Its stress–strain curves revealed a clearly defined elastic domain, followed by a sharp fracture. At higher testing speeds, a natural transition toward semi-ductile behavior was observed, with strains exceeding 4% and a significantly increased energy absorption capacity (~106 J), indicating remarkable adaptability to dynamic loading conditions without compromising structural integrity.

The Q-g composite (epoxy matrix without Coremat Xi3), although based on a stiffer resin, exhibited a more brittle trend. The maximum tensile strength remained slightly below that of Q-c (up to ~243 MPa at 1000 mm/min, also with low variation with test rate), and the force–time curves showed abrupt failure events, with increasing variability between samples at higher speeds. Although the strain at break increased slightly under dynamic conditions, the lack of a clear plastic phase and the rapid crack propagation indicated high sensitivity to structural imperfections and reduced energy dissipation capacity.

By contrast, the introduction of the Coremat Xi3 interlayer produced essential changes in the tensile response, enhancing the deformability and enabling a more progressive failure in QM-c and QM-g. The QM-c composite (polyester matrix with Coremat Xi3) showed reduced average strength (maximum 182 MPa), but a noticeable improvement in ductility with increasing test speed, reaching strains of up to 3.6% and energy absorption around 78 J. This transition is associated with a progressive failure mechanism in which the Coremat Xi3 layer functions as a compliant zone that redistributes stress and delays crack propagation.

The most noteworthy case was the QM-g composite (epoxy resin with Coremat Xi3), which combined the advantages of a rigid matrix with the benefits of an interlayer. Starting from a brittle response at 10 mm/min (strain at break being only ~2.2% and tensile strength ~174 MPa at 1000 mm/min), the material evolved into a remarkably deformable structure under high-speed loading, reaching strain at break values above 4% at 1000 mm/min and bearing an energy at break of ~87 J. Its curves for stress, strain, and energy with time indicated diffuse and progressive failure, with delayed structural collapse, confirming the Coremat Xi3′s role in attenuating crack growth and facilitating gradual energy dissipation through local micro-deformation.

Regarding stiffness, the highest Young’s modulus values were recorded for the epoxy-based systems. However, this came at the expense of flexibility and a stronger tendency toward brittle failure. Polyester-based composites, though slightly less stiff, compensated for a more favorable evolution of ductility and more stable performance under dynamic loading.

Taken together, these results highlight that each structural configuration provides specific advantages depending on the application’s requirements. Q-c is ideal for applications requiring high stiffness and long-term strength retention. Q-g may be used in scenarios where immediate stiffness is needed but requires careful control of defects. Meanwhile, QM-c and especially QM-g are recommended for situations demanding enhanced energy absorption, ductility, and progressive failure under tensile dynamic loads.

Ultimately, the tensile behavior of these composites is governed by a complex interaction among matrix type, structural architecture, and test rate.

Moreover, the results presented herein are particularly relevant for marine applications, where composites are frequently subjected to variable tensile loads, cyclic fatigue, and dynamic stresses, caused by wave motion, impact, or high-speed operation. The ability of materials, such as QM-g and QM-c to absorb energy, exhibit controlled deformation, and maintain structural integrity under high strain rates positions them as promising candidates for structural elements in shipbuilding, offshore platforms, and marine renewable energy systems. The high stiffness and consistent tensile performance of Q-c also make it suitable for semi-static marine structures requiring dimensional stability and strength over time. Thus, the comparative tensile testing conducted in this study provides critical insights into the selection and optimization of composite materials tailored for demanding marine environments.

## Figures and Tables

**Table 1:** Relevant references for tensile tests for composite with glass fibers reinforcement.

First Author (Year)	Composite (Glass Fiber Reinforcement, Resin, Additives)	Specimen Dimensions	Test Rate	Ultimate Tensile Strength (MPa)	Young’s Modulus (GPa)	Energy at Break (J)	Strain at Break (%)
Ou, Y. (2016) [20]	Unidirectional GFRP, epoxy	ASTM D3039	25–200 s^−1^	700–900	40–50	Not reported	1.5–2.0
Cui, J. (2019) [21]	Thermoplastics matrix with long glass fibers	Not specified	0.001–400 s^−1^	75.4–146.7	5.2–7.3	Not reported	2.8–4.6
Chiracu, I.G. (2024) [22]	Composite made of quadriaxial glass fiber fabric, epoxy matrix	8 layers	10–1000 mm/min (bending)	502–562	20–23	Not reported	3.31–3.61
		16 layers		484–546	17.8–19.2		3.5–4.0
				Not sensitive to test rate			Not sensitive to test rate
Shahzad, A. (2011) [23]	Chopped strand mat (CSM) GFRP polyester matrix	Not specified	Not specified	200–250	10–15	Not reported	1.5–2.0
Zhang, X. (2022) [24]	Unidirectional GFRP, epoxy	ASTM D3039	6 × 10^−5^ to 260 s^−1^	500–800	35–45	Not reported	1.8–2.5
Mahato, K.K. (2016) [25]	GFRP with epoxy matrix and MWCNTs	Not specified	1 mm/min	UTS increases with 15.11% adding 0.1% MWCNT	Not specified	Not reported	Not specified
Bochnia, J., (2020) [26]	PA 3200 GF (polyamide + 30% glass fibers)	80 × 10 × 4 mm	Not specified	48–53	Not specified	Not specified	Not specified
Ramesh, M. (2013) [27]	Glass fibers in epoxy matrix	250 × 25 × 2.5 mm	0.0017 s^−1^ to 160 s^−1^	200–350	20–30		1.5–2.5
Dong, C. (2013) [28]	Glass fiber or carbon fibers in epoxy matrix	250 × 25 × 2 mm	5 mm/min	300–600	25–60		1.2–2.0
Ismail, K.I. (2023) [29]	PLA reinforced with glass fibers (3D printing)	165 × 13 × 3.2 mm	Not specified	45–60	2.5–3.0		2.5–3.5
Stanciu, M.D., (2021) [30]	GFRP-RT500 (4 layers 0/90)	250 × 25 × 2 mm	1 mm/min	400–450	21–22		1.8–2.2
	GFRP-MAT450 (short fibers)		1 mm/min	200–250	10–11		1.0–1.5
	GFRP-RT500 (4 layers 0/90)		20 mm/min	380–430	18–19		1.6–2.0
	GFRP-MAT450 (short fibers)		20 mm/min	180–230	9–10		0.9–1.4

**Table 2:** Calculated strain rate for the tested composites.

Crosshead Speed (Test Rate)		$\mathrm{Strain}\;\mathrm{Rate},\;\dot{\varepsilon }$ = v/L_0_
(mm/min)	[mm/s)	(s^−1^)
10	0.167	0.0033
200	3.33	0.0666
500	8.33	0.1666
1000	16.67	0.3334

**Table 3:** Failure modes—influence of the Coremat^®^ Xi 3 layer.

Composite (Resin)	Test Rate (mm/min)	Without Coremat Xi3 Layer (Q-g and Q-c)	With Coremat Xi3 Layer (QM-g and QM-c)
Epoxy (coded g)	10	Controlled pull-out, good resin quality	Pull-out + bending in Coremat area
	200	Fiber pull-out + reduced delamination	Localized deformation in Coremat layer
	500	Pull-out + controlled crack propagation	Mixed failure: pull-out + interlaminar cracking
	1000	Large delaminations among several layers	Larger delaminations among layers; different aspect of the broken samples; visible successive rupture of the layers (see last photo in line for QM-g, Figure 2)
Polyester (coded c)	10	Pronounced brittleness, clean cracks	Gradual failure, stress diffusion
	200	Pull-out + interfacial debonding	Ductile failure, controlled delamination
	500	Massive, uncontrolled delamination	Layered crack propagation
	1000	Extensive delamination across layers, pronounced fragmentation across several layers, fiber pull-out	Large-scale layer separation, gradual failure, visible fiber bridging

**Table 4:** Failure modes—influence of test rate on failure mode for each composite.

Composite	Test Rate			
	10 mm/min	200 mm/min	500 mm/min	1000 mm/min
Q-g (epoxy resin, without Coremat Xi3)	Controlled pull-out, tough resin, smaller areas with delamination	Fiber pull-out, moderate delamination	Extensive matrix cracking, brittle zones appear	Extensive matrix cracks, multiple delaminations, brittle failure with sudden fiber breakage across several layers
QM-g (epoxy resin, with Coremat Xi3)	Pull-out + bending in Coremat area	Localized deformation in intermediate layer	Extensive delamination, successive breaks of fibers in different layers, throughout the sample, and in the loading direction	Mixed failure continues, more dispersed damage through Coremat
Q-c (polyester resin, without Coremat Xi3)	Brittle fracture, clean cracks	Pull-out + interfacial debonding		Severe fragmentation, high brittleness, abrupt failure
QM-c (polyester resin, with Coremat Xi3)	Gradual failure, efficient energy dissipation	Controlled delamination, partial pull-out		Energy absorption visible, layered cracks with fiber bridging

**Table 5:** Comparing values of several mechanical characteristics of resins.

Characteristic	Enydyne H 68372 TA [66]	SikaBiresin CH80-2 [69]
Density (at 20 °C), g/cm^3^	1.10	1.14
Tensile strength (ISO 527) [51], MPa	45	90
Strain at break, %	1.5	5.6%
Flexural strength (ISO 178 [52]), MPa	65	130
Flexural modulus, MPa	3300	3000

**Table 6:** Comparing data for the tested composites at 10 mm/min and 1000 mm/min.

Characteristic	Formula	Q-c	Q-g	QM-c	QM-g
Young’s modulus, *E*	${(E}_{\left(1000\right)}-E_{\left(10\right)})\times 100/E_{\left(10\right)}\;\left[\%\right]$	30.06	33.42	41.20	11.65
$F_{\max}$	${{(F}_{\max}}_{\left(1000\right)}-F_{\max\left(10\right)})\times 100/F_{\max\left(10\right)\;}\left[\%\right]\;$	8.48	−6.73	−3.86	0.01
Tensile strength, $\sigma_{\max}$	${{(\sigma }_{\max}}_{\left(1000\right)}-{\sigma_{\max}}_{\left(10\right)})\times 100/{\sigma_{\max}}_{\left(10\right)}\;\left[\%\right]$	6.25	1.10	−1.40	10.97
Strain at break, $\varepsilon_{r}$	${{(\varepsilon }_{r}}_{\left(1000\right)}-{\varepsilon_{r}}_{\left(10\right)})\times 100/{\varepsilon_{r}}_{\left(10\right)}\;\left[\%\right]\;\;$	111.11	56.57	27.38	85.45
Energy at break, $E_{r}$	${{(E}_{r}}_{\left(1000\right)}-{E_{r}}_{\left(10\right)})\times 100/{E_{r}}_{\left(10\right)}\;\left[\%\right]\;$	153.37	93.34	97.91	152.02

**Figure A1:**
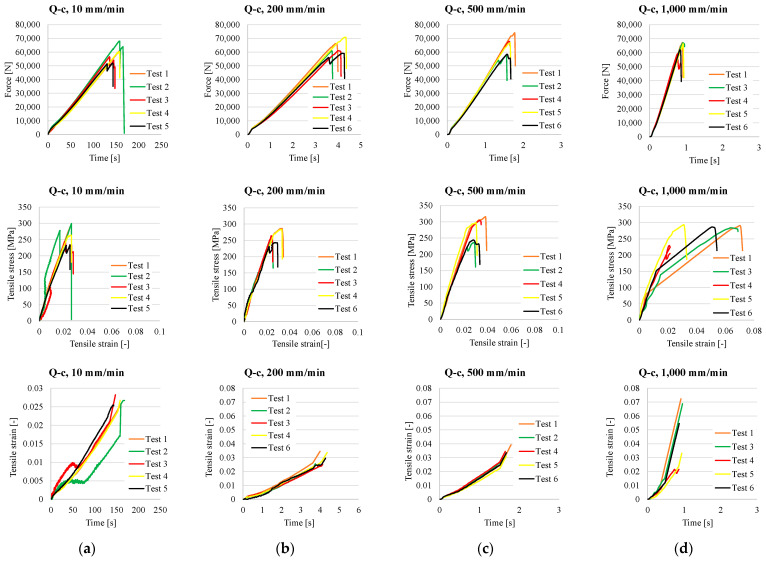
The force–time curves, tensile stress–tensile strain, and tensile strain–time curves for composite Q-c for five tensile tests at different test rates: (**a**) 10 mm/min, (**b**) 200 mm/min, (**c**) 500 mm/min, (**d**) 1000 mm/min.

**Figure A2:**
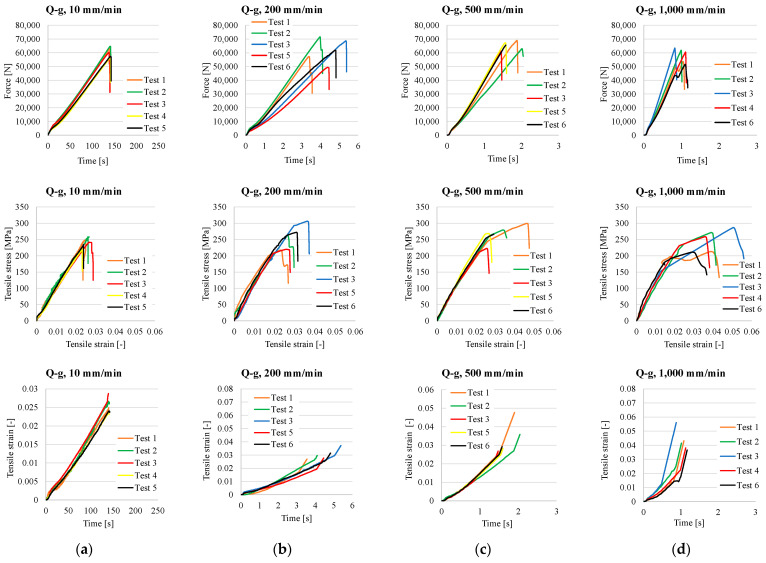
The force–time curves, tensile stress–tensile strain, and tensile strain–time curves for composite Q-g for five tensile tests at different test rates: (**a**) 10 mm/min, (**b**) 200 mm/min, (**c**) 500 mm/min, (**d**) 1000 mm/min.

**Figure A3:**
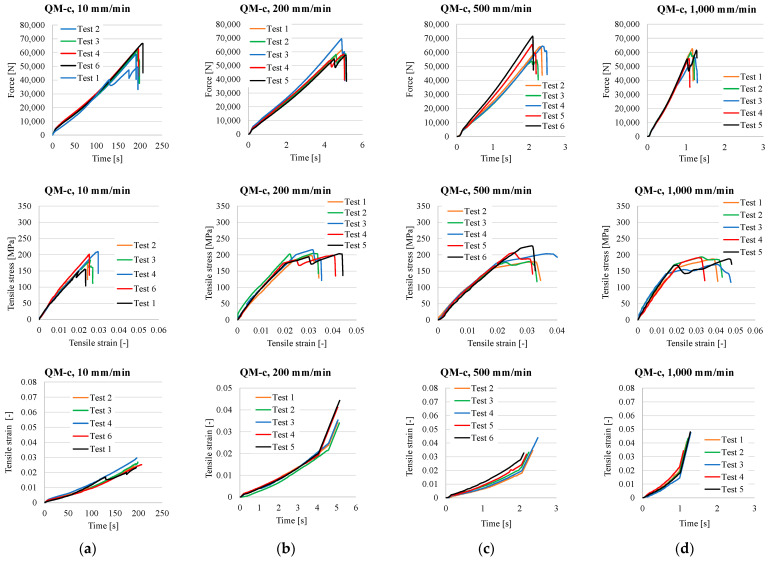
The force–time curves, tensile stress–tensile strain, and tensile strain–time curves for composite QM-c for five tensile tests at different test rates: (**a**) 10 mm/min, (**b**) 200 mm/min, (**c**) 500 mm/min, (**d**) 1000 mm/min.

**Figure A4:**
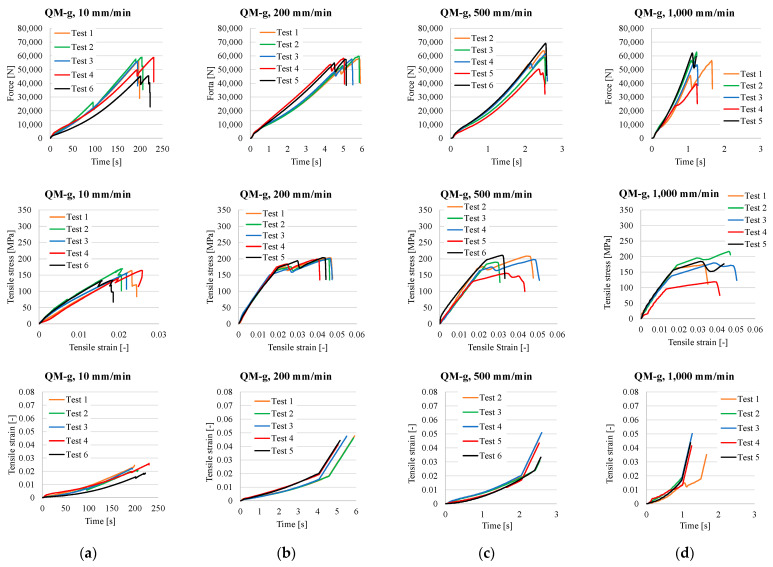
The force–time curves, tensile stress–tensile strain, and tensile strain–time curves for composite QM-g for five tensile tests at different test rates: (**a**) 10 mm/min, (**b**) 200 mm/min, (**c**) 500 mm/min, (**d**) 1000 mm/min.

## Data Availability

Data are contained within the article.

## Appendix A

Figure A1 presents the force–time, tensile stress–tensile strain, and tensile strain–time curves for composite Q-c, at different test rates, for five tests at each test rate. Each set of curves reveals two distinct regions. The first region, where the curves from the five tensile tests are closely aligned, suggests a linear relationship that can be effectively modeled using linear regression, making it suitable for engineering design purposes. The second region displays greater scatter among the curves, indicating that this part of the loading process contributes to energy absorption during failure, but the material exhibits non-predictable behavior. Consequently, this region represents a less predictable mechanical response of the composite material.

The Q-c composite displays a robust and highly reproducible tensile behavior across all tested rates (10–1000 mm/min). The force–time and stress–strain curves show a clearly defined almost linear elastic response, followed by a sudden failure with no evident plastic plateau or yielding zone. This response is typical for well-compacted tough composites with strong fiber–matrix bonding.

At 10 mm/min, the stress–strain curves reveal a steep elastic slope (high Young’s modulus), with maximum stress values around 260 MPa and relatively low strains at failure (~2.5–3%). Except for test 2, all tests overlap closely, confirming excellent structural homogeneity and uniform controllable load transfer by a simpler relationship.

As the test rate increases to 200 mm/min and 500 mm/min, the maximum stress slightly increases (~270–280 MPa), and the strain at break becomes more pronounced (~3–3.5%). This suggests good adaptability of the polyester matrix under dynamic loading, maintaining structural integrity and dissipating stresses more efficiently.

At 1000 mm/min, the composite exhibits a clear transition to a more ductile response, with strains at break exceeding 4%. While the maximum stress remains similar, the energy at break increases significantly, as shown by the enlarged area under the stress–strain curve. Deformation occurs more gradually, indicating potential mechanisms like local delaminations or progressive fiber–matrix debondings. The strain–time behavior evolves predictably, from slow, steady growth at low test rates to rapid elongation at high rates. This characterizes a semirigid material with good tensile stiffness and increasing deformation capacity under fast loading.

In conclusion, Q-c demonstrates excellent tensile behavior, marked by high stiffness, consistent strength, and a clear transition from brittle to semiductile with increasing test speed. These features make it suitable for structural applications under dynamic axial loads, where both load-bearing capacity and deformation tolerance are critical.

The Q-g composite (Figure A2), consisting of a rigid epoxy matrix and quadriaxial reinforcement, without any intermediate layer, displays a brittle tensile behavior dominated by stiffness and abrupt failure. The force–time and stress–strain curves show a strong linear elastic phase followed by sudden fracture, with little to no plastic deformation.

At 10 mm/min, the stress–strain curves exhibit high stiffness (steep slope), with maximum stresses around 240–250 MPa and low failure strains (~2.5–2.8%). The rupture is clean and immediate, indicating limited internal energy dissipation and low ductility, typical of highly crosslinked epoxy systems. At 200 mm/min, maximum stress increases to approx. 255 MPa, and strain at break slightly improves (~3.0%). However, greater variability is observed between samples, suggesting sensitivity to internal defects or local delaminations. The force–time curves are steeper, reflecting fast energy release upon failure. At 500 mm/min, the tensile behavior becomes less uniform. Maximum stress remains stable (~250–260 MPa), but some curves show extended local failure zones, hinting at the activation of secondary mechanisms like interlaminar fracture or subcritical crack growth. At 1000 mm/min, the composite starts to show semiductile behavior, with strain at break exceeding 4% in some samples. While the maximum stress stays constant, absorbed energy density increases slightly, suggesting more progressive damage evolution. Fracture occurs faster.

Q-g demonstrates a stiff and strong response, but with limited ductility. Higher test rates promote better extensibility, but also increase variability, indicating a degree of sensitivity to microstructural imperfections. The material lacks a significant plastic region, limiting its energy absorption capacity compared to polyester-based systems.

The QM-c composite (Figure A3) exhibits a tensile behavior with reduced stiffness, but enhanced deformability due to the structural influence of the intermediate layer.

At 10 mm/min, the stress–strain curves display a gentler initial slope, reflecting a lower Young’s modulus. Maximum tensile stress is reduced (~185–190 MPa), but strain at break increases (~2.5–2.6%). The presence of Coremat Xi3 introduces a locally compliant region that allows for additional deformation, while slightly lowering overall strength. At 200 mm/min, both strength (~195–200 MPa) and strain at break (~3%) improve. The intermediate layer appears to promote more uniform stress distribution, delaying the onset of damage and enabling smoother failure progression. At 500 mm/min, the material reaches a favorable balance between strength and ductility, with consistent maximum stress (~200 MPa) and higher deformation (~3.5%). The force–time and strain–time curves show extended durations to failure and more gradual fracture propagation. At 1000 mm/min, a slight decrease in tensile strength (~182 MPa) is observed, but strain at break continues to increase. Force–time curves exhibit fluctuations during failure, suggesting localized collapse in the Coremat Xi3 zones, which allow for energy dissipation and crack deflection.

In summary, the composite QM-c exhibits lower stiffness than the composite Q-c, but significantly higher ductility, especially at high test rates. The Coremat Xi3 layer enhances energy absorption and promotes a more controlled failure mechanism, making this composite suitable for structural applications subject to dynamic tensile loads.

The composite QM-g (Figure A4) has a tensile response that balances rigidity with progressive deformation capacity. The behavior varies notably with test rate, synergically influenced both by the resin type and the intermediate layer.

At 10 mm/min, the material behaves in a brittle manner. The stress–strain curves show high stiffness, but reduced tensile strength (~175–180 MPa) and low strain at break (~2.2%). The Coremat Xi3′s contribution is limited at low test rate, and fracture occurs suddenly with minimal post-elastic (plastic) behavior. At 200 mm/min, tensile strength increases (~200 MPa), and strain at break reaches ~3.3–3.4%. A slight post-peak stretch appears, indicating that Coremat Xi3 begins to affect the failure mechanism, redistributing local stresses and enabling a more progressive fracture. At 500 mm/min, ultimate strength (~205 MPa) and strain (~3.6%) stabilize. Curves show a clear delay in rupture onset and more uniform failure evolution, confirming that the composite adapts well to intermediate loading rates. At 1000 mm/min, the composite QM-g performs better: strain at break exceeds 4%, and energy absorption improves markedly. Stress–strain curves display larger areas under the curve (the strain energy density), suggesting controlled damage progression. Force–time plots show extended failure durations, signaling that Coremat Xi3 efficiently delays crack propagation and enables micro-deformation mechanisms to develop.

In conclusion, the composite QM-g achieves superior dynamic performance compared to composite Q-g. While initially brittle at low test rate, the presence of Coremat Xi3 enhances ductility and energy dissipation at higher test rates. This makes the composite QM-g highly suitable for demanding applications where both stiffness and toughness under dynamic tensile loads are essential.

## Appendix B

Table A1 presents the dimensions of the tested samples. Tests with extreme values were eliminated. It is worthy to mention that SD% (standard deviation as percentage from the average value) is under 10% for all sets of measurements.

**Table A1:** Dimensions of the samples tested in traction (five tests, the test results are presented in Appendix A for each composite).

**10 mm/min**															
**Q-c**				**Q-g**				**QM-c**				**QM-g**			
**Test no.**	**Specimen label**	**Thickness**	**Width**	**Test no.**	**Specimen label**	**Thickness**	**Width**	**Test no.**	**Specimen label**	**Thickness**	**Width**	**Test no.**	**Specimen label**	**Thickness**	**Width**
		**(mm)**				**(mm)**				**(mm)**				**(mm)**	
1	Q-c_1	7.70	27.72	1	Q-g_1	8.92	28.40	1	QM-c_1	11.79	26.95	1	QM-g_1	12.50	27.70
2	Q-c_2	8.36	29.21	2	Q-g_2	9.11	27.53	2	QM-c_2	12.34	26.54	2	QM-g_2	12.44	27.98
3	Q-c_3	8.65	26.81	3	Q-g_3	9.20	27.18	3	QM-c_3	12.19	27.47	3	QM-g_3	12.79	27.94
4	Q-c_4	8.71	26.25	4	Q-g_4	9.21	27.41	4	QM-c_4	11.91	25.15	4	QM-g_4	12.12	29.58
5	Q-c_5	8.64	25.69	5	Q-g_5	9.12	26.81	6	QM-c_21	11.82	26.20	6	QM-g_21	12.37	27.52
Average		8.41	27.14	Average		9.11	27.47	Average		12.01	26.46	Average		12.44	28.14
SD		0.42	1.38	SD		0.12	0.59	SD		0.24	0.87	SD		0.24	0.82
SD%		5.00	5.09	SD%		1.28	2.15	SD%		2.02	3.30	SD%		1.94	2.93
200 mm/min															
Q-c				Q-g				QM-c				QM-g			
Test no.	Specimen label	Thickness	Width	Test no.	Specimen label	Thickness	Width	Test no.	Specimen label	Thickness	Width	Test no.	Specimen label	Thickness	Width
		(mm)				(mm)				(mm)				(mm)	
1	Q-c_6	8.40	27.51	1	Q-g_6	9.03	29.19	1	QM-c_6	11.95	25.89	1	QM-g_6	10.84	26.35
2	Q-c_7	8.88	27.83	2	Q-g_7	9.32	29.33	2	QM-c_7	11.40	25.13	2	QM-g_7	10.79	27.89
3	Q-c_8	8.64	26.78	3	Q-g_8	8.11	27.65	3	QM-c_8	11.82	27.18	3	QM-g_8	10.55	27.11
4	Q-c_9	8.52	29.36	4	Q-g_9	7.26	28.83	4	QM-c_9	11.52	25.37	4	QM-g_9	10.90	26.76
5	Q-c_10	8.45	29.01	5	Q-g_10	8.21	27.33	5	QM-c_10	11.41	24.76	5	QM-g_10	11.34	27.00
Average		8.58	28.10	Average		8.39	28.47	Average		11.62	25.67	Average		10.88	27.02
SD		0.19	1.07	SD		0.82	0.87	SD		0.25	0.94	SD		0.29	0.57
SD%		2.23	3.81	SD%		9.73	3.06	SD%		2.16	3.66	SD%		2.64	2.09
500 mm/min															
Q-c				Q-g				QM-c				QM-g			
Test no.	Specimen label	Thickness	Width	Test no.	Specimen label	Thickness	Width	Test no.	Specimen label	Thickness	Width	Test no.	Specimen label	Thickness	Width
		(mm)				(mm)				(mm)				(mm)	
1	Q-c_11	8.12	28.87	1	Q-g_11	8.33	27.61	2	QM-c_12	12.02	28.86	2	QM-g_12	10.85	28.19
2	Q-c_12	8.73	27.99	2	Q-g_12	8.31	27.12	3	QM-c_13	11.68	26.03	3	QM-g_13	11.05	28.14
4	Q-c_14	8.11	25.53	3	Q-g_13	9.69	28.36	4	QM-c_14	11.66	27.12	4	QM-g_14	10.90	28.55
5	Q-c_15	8.31	27.02	5	Q-g_15	9.18	27.30	5	QM-c_15	11.45	27.67	5	QM-g_15	11.61	27.87
6.	Q-c_22	8.80	27.21	6	Q-g_22	9.23	26.73	6	QM-c_23	12.01	26.12	6	QM-g_23	12.14	27.01
Average		8.41	27.32	Average		8.95	27.42	Average		11.76	27.16	Average		11.31	27.95
SD		0.33	1.24	SD		0.61	0.61	SD		0.25	1.17	SD		0.55	0.58
SD%		3.94	4.54	SD%		6.78	2.23	SD%		2.09	4.32	SD%		4.90	2.07
1000 mm/min															
Q-c				Q-g				QM-c				QM-g			
Test no.	Specimen label	Thickness	Width	Test no.	Specimen label	Thickness	Width	Test no.	Specimen label	Thickness	Width	Test no.	Specimen label	Thickness	Width
		(mm)				(mm)				(mm)				(mm)	
1	Q-c_16	8.47	27.21	1	Q-g_16	9.08	27.67	1	QM-c_16	11.86	28.09	1	QM-g_16	11.04	29.44
3	Q-c_18	8.42	28.01	2	Q-g_17	8.25	27.59	2	QM-c_17	11.70	26.40	2	QM-g_17	10.94	26.51
4	Q-c_19	8.82	29.20	3	Q-g_18	8.19	26.96	3	QM-c_18	11.79	28.00	3	QM-g_18	12.17	25.60
5	Q-c_20	8.43	26.81	4	Q-g_19	8.33	28.03	4	QM-c_19	11.81	24.60	4	QM-g_19	11.86	28.23
6	Q-c_23	8.24	26.24	6	Q-g_23	8.97	27.22	5	QM-c_20	11.76	27.80	5	QM-g_20	12.07	27.91
Average		8.48	27.49	Average		8.56	27.49	Average		11.78	26.98	Average		11.62	27.54
SD		0.21	1.15	SD		0.43	0.41	SD		0.06	1.50	SD		0.58	1.50
SD%		2.50	4.19	SD%		4.97	1.51	SD%		0.50	5.54	SD%		5.02	5.46

SD—standard deviation of the characteristics for five values; SD% is the standard deviation in percentage, SD% = SD × 100/Average.

Table A2 presents information of the composite characteristics. The same calculations were carried out for the plate used in [35].

The fiber volume fraction, V_f_, is calculated as follows:

- For composites without Coremat Xi3, the composite mass is
  V_composite_ × ρ_composite_ = V_f_ × ρ_f_ + m_resin_ (A1)
- For composites with Coremat Xi3, the composite mass is
  V_composite_ × ρ_composite_ = V_f_ × ρ_f_ + m_mat_ + m_resin_ (A2)

with V_composite_ = l^2^·h_average,_ l being the size of the square plate (considering l = 300 mm after finishing the plate edges), ρ_composite_ is the composite density, and h_average_ being the average thickness of the plate. Here, ρ_f_ is the fiber density (considered as ρ_f_ = 2540 kg/m^3^ for glass fibers). The fiber volume ratio is expressed as follows:

V_f/c_ = V_f_/V_composite_ (A3)

From Table A2, the fiber fraction volume, calculated for the plate from which the tensile sample were cut, exhibits an average value of 0.442 for the composite Q-c and 0.441 for the composite Q-g. For the composites with Coremat Xi3 middle layer, this characteristic is 0.330 for QM-c and 0.339 for QM-g. The layer of Coremat Xi3 reduces the volume fraction of glass fibers in the composite by 25.3% as compared to the fiber volume fraction of the composite without mat for the composites using the epoxy resin. For composites with unsaturated polyester resin, the fiber volume fraction is reduced with 23.12%.

These data in Table A2 are for plates of 300 mm × 300 mm, polished on their edges. From each plate, we could cut 8 samples having the width in the range 25–29 mm (this range is caused by the cutting edge of the cutting disk; the plate edges were eliminated; the actual values for height and width of each sample were introduced in test machine software). Thus, from each set of 3 plates (with the same materials and technology), there were cut 24 samples. For four values of the test rate, we need 4 × 5 samples = 20 samples (at least) for each test and composite.

**Table A2:** Characteristics of the produced composite plates.

Plate	Fabric Mass, m_f_	Composite Plate Mass	Resin Mass *	Mass Fiber Ratio (m_f_/m_composite_)	Surface Density (m_composite_/A_composite **_)	V_f/_V_composite_	h_1_	h_2_	h_3_	h_4_	h_average_
	(g)	(g)	(g)		(kg/m^2^)		(mm)	(mm)	(mm)	(mm)	(mm)
0	1	2	3	4	5	6	7	8	9	10	11
Q-c-02	855.0	1443.7	588.672	0.592	16.041	0.416	8.75	9.10	8.90	9.20	8.988
Q-c-03	865.5	1461.4	595.901	0.639	14.967	0.470	8.20	7.95	7.80	8.30	8.063
Q-c-04	863.0	1457.2	594.180	0.608	15.250	0.440	8.65	8.40	8.70	8.55	8.575
Average Q-c	861.2	1454.1	592.918	0.613	15.419	0.442	8.53	8.48	8.46	8.68	8.542
Q-g-07	883.0	1295.6	412.583	0.682	13.939	0.462	8.40	8.45	8.35	8.25	8.363
Q-g-08	850.0	1247.2	397.164	0.625	15.017	0.431	8.65	8.70	8.60	8.55	8.625
Q-g-09	848.5	1245.0	396.463	0.651	14.444	0.430	8.60	8.55	8.65	8.75	8.638
Average Q-g	860.5	1262.6	402.070	0.653	14.467	0.441	8.55	8.56	8.53	8.51	8.542
QM-c-08	823.0	1518.1	687.108	0.547	17.278	0.313	11.65	11.20	11.40	11.70	11.488
QM-c-09	866.6	1598.5	724.433	0.558	17.044	0.343	10.90	11.10	10.75	11.45	11.050
QM-c-10	852.5	1572.5	712.024	0.552	17.144	0.333	10.90	11.45	11.25	11.25	11.213
Average QM-c	847.4	1563.1	707.855	0.552	17.155	0.330	11.15	11.25	11.13	11.46	11.250
QM-g-07	850.3	1392.6	534.309	0.617	15.122	0.339	11.25	11.55	10.35	10.80	10.988
QM-g-08	864.0	1415.0	542.547	0.635	15.139	0.344	11.20	10.70	11.25	10.75	10.975
QM-g-09	845.5	1384.7	532.248	0.642	15.033	0.334	11.20	11.10	10.90	11.10	11.075
Average QM-g	853.3	1397.5	536.368	0.631	15.098	0.339	11.21	11.11	10.83	10.88	11.013

* m_resin_ = m_composite_ − m_fabric_ (meaning Column 2–Column 3) for composites Q-c and Q-g and m_resin_ = m_composite_ − m_fabric_ − m_mat for the composites QM-c and QM-g_; m_mat_ ≅ 8 g for each layer of 300 mm × 300 mm; ** A_composite_ ~ 0.09 m^2^.
